# Unraveling TGF-β1’s Role in Mediating Fibrosis and Cell Death in Feline Kidney Cells

**DOI:** 10.3390/ani15020257

**Published:** 2025-01-17

**Authors:** Chanyanuch Intachat, Phongsakorn Chuammitri, Benjaporn Sornpet, Prapas Patchanee, Nawin Manachai, Kakanang Piyarungsri

**Affiliations:** 1 Faculty of Veterinary Medicine, Chiang Mai University, Chiang Mai 50100, Thailand; intachat.c@gmail.com (C.I.); phongsakorn.c@cmu.ac.th (P.C.); prapas.pat@cmu.ac.th (P.P.); nawin.m@cmu.ac.th (N.M.); 2 Research Center of Producing and Development of Products and Innovation for Animal Health and Production, Chiang Mai University, Chiang Mai 50100, Thailand; 3 Center of Veterinary Medical Diagnostic and Animal Health Innovation, Chiang Mai University, Chiang Mai 50100, Thailand; benjaporn.s@cmu.ac.th

**Keywords:** Bcl-2, cat, chronic kidney disease, mitogen-activated protein kinase, transforming growth factor-β1

## Abstract

Chronic kidney disease (CKD) is prevalent among older cats. The transforming growth factor beta 1 (TGF-β1) pathway is associated with renal fibrosis. TGF-β1 signaling through the non-canonical/smad-independent pathway activates mitogen-activated protein kinase (MAPK) signaling, which is linked to fibrosis and apoptosis. The MAPK pathway regulates the Bcl-2 protein family, which is known for its anti-apoptosis properties. This study aimed to quantify the mRNA expression of the *TGFβ*, *MAPK*, and *Bcl2* genes and the protein expression of TGF-β1 and MAPK in feline kidney cells and tissue. A gene expression analysis was conducted using qPCR to calculate the relative gene expression, while the protein expression was assessed through Western blot analysis. Immunohistochemistry staining of TGF-β1 and MAPK was performed on feline kidney tissue. The results revealed the significant upregulation of *TGFβ* (*p* = 0.001) and considerable downregulation of *Bcl2* (*p* = 0.010) in doxorubicin-treated feline kidney cells. The immunostaining levels of TGF-β1 and MAPK were higher in the kidney tissue of cats with CKD than in non-CKD cats. However, there was no difference in *TGFβ*, *MAPK*, or *Bcl2* gene expression in CKD vs. non-CKD cats. The findings suggest that TGF-β1 and Bcl-2 are associated with renal fibrosis and apoptosis in feline kidney cells. A deeper understanding of the TGF-β1 pathway could enable veterinarians to monitor disease progression and mitigate complications in feline CKD.

## Simple Summary

Chronic kidney disease (CKD) is associated with fibrosis and apoptosis. Transforming growth factor beta 1 (TGF-β1) serves as a pro-fibrotic mediator, while mitogen-activated protein kinases (MAPKs) are related to fibrosis and apoptosis. TGF-β activates MAPK signaling; this regulates the Bcl-2 protein family, which is known for its anti-apoptosis properties. This study aimed to evaluate the levels of *TGFβ*, *Bcl2*, and *MAPK* mRNA expression in doxorubicin-treated feline kidney cells and naturally occurring CKD-affected kidney tissues, as well as determine the protein expression of TGF-β1 and MAPK in doxorubicin-treated feline kidney cells. The results indicate a significant increase in *TGFβ* and a considerable decrease in *Bcl2* in the feline kidney cells with doxorubicin-induced cytotoxicity. The protein expression of TGF-β1 and MAPK was numerically higher but not statistically different in both the feline kidney cells with doxorubicin-induced cytotoxicity and the kidney tissue of cats with CKD. This study concludes that TGF-β1 and Bcl-2 may be associated with renal fibrosis and apoptosis in feline kidney cells. In vivo, TGF-β1 and MAPK might be associated with renal fibrosis and apoptosis. Further research on these mediators could lead to the development of therapeutic medications that delay the progression of CKD.

## 1. Introduction

Chronic kidney disease (CKD) exhibits significant prevalence in cats. CKD is defined as an abnormality in the anatomy or function of one or both kidneys that persists for at least three months [1]. The prevalence of feline CKD differs in various countries, with rates of 3.6% in the United Kingdom [2]; 2.37% in Chiang Mai, Thailand [3]; and 50% in the United States [4]. Moreover, the prevalence of CKD in cats increases with age; 80.9% of cats affected by CKD are between 15 and 20 years old [4]. The most common lesion type associated with feline CKD is tubulointerstitial lesions, including interstitial fibrosis, interstitial inflammation, tubular mineralization, and hyperplastic arteriolosclerosis. Interstitial fibrosis is positively correlated with the progression of CKD in cats [5]. In the feline renal fibrosis process, several major pro-fibrotic mediators are involved, including TGF-β, transglutaminase 2 (TG2), endothelin 1 (ET1), and the renin-angiotensin-aldosterone system (RAAS).

The most crucial pro-fibrotic mediator is believed to be TGF-β1, which transforms various types of cells into myofibroblasts [6]. Transforming growth factor beta (TGF-β) plays a role in regulating cell proliferation, serving as either an inhibitor or stimulator, depending on the signaling pathways. In addition, TGF-β is involved in wound healing, the differentiation of cells, and the immune system. It is also associated with pathological processes, including connective tissue disorders, fibrosis, and cancer [7]. TGF-β signaling consists of two pathways: the smad-dependent/canonical pathway and the smad-independent/non-canonical pathway [8]. TGF-β is associated with apoptosis through these two pathways: the smad-dependent pathway affects BCL-2 family expression, and the smad-independent pathway induces apoptosis by activating the p38 and JNK pathways [9]. In the context of cats, a previous study demonstrated that the urinary TGF-β1/creatinine ratio was significantly increased in CKD cats compared to healthy cats and was positively correlated with the level of creatinine in the serum [10]. A study examining feline kidney tissue revealed that the level of TGF-β1 immunohistochemistry staining was higher in cats with chronic kidney disease (CKD) compared to healthy cats [11]. Interestingly, the concentration of TGF-β1 in the blood of CKD cats was significantly lower than that in healthy cats. Additionally, a previous study has suggested that low levels of circulating TGF-β1 and tubular atrophy with TGF-β1 immunoreactivity may be important for cats with CKD [12].

Mitogen-activated protein kinases (MAPKs) are enzymes that function as serine/threonine protein kinases. MAPKs are controlled by various extracellular stimuli, which in turn trigger gene expression, cell division, and cell survival. The MAPK pathway contributes to acute kidney injury (AKI) and CKD [13]. Previous studies have indicated that there is an association between the MAPK pathway and renal fibrosis in mice [14,15]. In mice, renal artery stenosis (RAS) surgery was used to induce tubular atrophy and interstitial fibrosis. The results revealed increased p38 MAPK signaling in mice undergoing RAS surgery compared to sham mice. Moreover, blocking p38 MAPK with inhibitors in RAS mice led to a reduction in interstitial inflammation, interstitial fibrosis, and tubular atrophy compared to the use of RAS without p38 MAPK inhibitors [14]. Furthermore, the MAPK pathway is involved in the apoptosis process, with the p38 MAPK and JNK pathways regulating the Bcl2 family [16]. The B-cell lymphoma 2 (Bcl-2) protein family plays a role in cell growth and apoptosis regulation. It has been found to be associated with renal cell death during kidney injury [17]. There are three classes within the Bcl-2 protein family: anti-apoptosis (Bcl-XL, Bcl-2), pro-apoptosis (Bak, Bax), and BH3-only proteins (Bid, Bad) [18]. In a study, the Bcl-2 expression in the blood of CKD cats was significantly lower than that in healthy cats. In feline renal tissue, Bcl-2 immunoreactivity was found within glomerular and tubular epithelium cells. Previous studies have suggested that Bcl-2 is an acceptable diagnostic marker for feline CKD. Bcl-2 downregulation may play a role in the progression of feline CKD [19].

However, there are few studies on the TGF-β1 signaling pathway, which involves MAPKs and Bcl-2, in cats with chronic kidney disease. Therefore, in this study, we aimed to evaluate the *TGFβ*, *MAPK*, and *Bcl2* gene expression in the context of doxorubicin-induced cytotoxicity in feline kidney cells and the kidney tissue of cats with naturally occurring CKD. We also aimed to determine the TGF-β1 and MAPK protein expression in doxorubicin-treated feline kidney cells. We hypothesized that doxorubicin-induced cytotoxicity in feline kidney cells and the kidney tissue of cats with chronic kidney disease (CKD) would lead to increased levels of TGF-β1 and MAPK and decreased levels of Bcl-2.

## 2. Materials and Methods

### 2.1. Samples

#### 2.1.1. Feline Kidney Cell Line

The Cradle-Rees Feline Kidney (CRFK) cell line was procured from ATCC^®^ (CCL-94), Manassas, VA, USA, LOT 60980362. These cells originated from the cortex epithelium in the kidneys of 12-week-old female domestic cats. The cells were grown in Dulbecco’s Modified Eagle Medium (DMEM), 5% fetal bovine serum (FBS), 100 µg/mL streptomycin, 100 units/mL penicillin, and non-essential amino acid solution and incubated at 37 °C in 5% CO_2_ in a humidified incubator. The cells used in this study were obtained in 125–132 passages.

#### 2.1.2. Kidney Tissue

Kidney tissue was collected from cats with no kidney lesions (*n* = 6) and cats with CKD (*n* = 6) that died at the Small Animal Hospital, Faculty of Veterinary Medicine, Chiang Mai University. The criteria for cats with normal kidneys included the following: no history of kidney disease, serum creatinine levels of less than 1.6 mg/dL, urine specific gravity greater than 1.035, and no histopathological lesions in the kidneys. The criteria for CKD cats included a history of kidney disease, serum creatinine levels greater than 1.6 mg/dL, urine specific gravity less than 1.035, and the presence of histopathological lesions such as those associated with tubulointerstitial fibrosis, tubular atrophy, and glomerulosclerosis. This study included both feral and owned cats, all of which died of natural causes, as well as one tissue sample obtained from a nephrectomy. A summary of the cats’ parameters is provided in Table 1 The samples were preserved in 10% formalin or frozen at −20 °C to maintain freshness. Paraffin block samples were prepared from 10% formalin-preserving tissue, cut into slides, and stained with hematoxylin and eosin. A pathologist examined the histological morphologies and categorized them as having no kidney lesions or exhibiting CKD. They were also used for immunohistochemistry. The frozen tissue samples were utilized to analyze the gene expression through qPCR and the protein expression using Western blotting. The human and animal experimentation protocol was approved by the Ethics Committee, Faculty of Veterinary Medicine, Chiang Mai University, under reference number S1/2566.

### 2.2. Sample Collection

#### 2.2.1. Cytotoxic Assay of Doxorubicin

The CRFK cells were incubated for different durations with different concentrations of doxorubicin (DOX) (catalog no. 324380). The concentrations of DOX used were 0, 1, 2, 4, and 8 µM, and the cells were incubated for 48, 72, and 96 h [20]. The CRFK cells were grown in a 96-well plate and kept overnight in a 37 °C and 5% CO_2_ humidified incubator before adding DOX. The cell viability was determined using the MTT colorimetric assay [21]. In this study, the appropriate dose and duration that could decrease the cell viability by 50% were applied.

#### 2.2.2. DOX-Induced Cytotoxicity Test

The CRFK cells were seeded into 6-well plates and then incubated at 37 °C in a humidified 5% CO_2_ incubator overnight. The appropriate dose and duration of DOX were 8 µM and 48 h, respectively. Next, DOX 8 µM was added to the CRFK cells, and they were incubated for 48 h. After this, the feline kidney cells with DOX-induced cytotoxicity were evaluated for the mRNA expression of *TGFβ*, *MAPK*, and *Bcl2*.

#### 2.2.3. RNA Extraction

RNA was harvested from the CRFK cells and kidney tissue. The CRFK cells were grown in a 6-well plate before harvesting the RNA. The RNA was extracted using the TRIzol^®^ (Sigma-Aldrich, St. Louis, MO, USA) reagent. First, the medium was removed; then, the TRIzol^®^ reagent was added to the plate. The extractant was then transferred to an Eppendorf tube and centrifuged at 4 °C and 12,000 rpm for 3 min. The supernatant was removed, and isopropanol was added. The mixture was then centrifuged at 4 °C and 12,000 rpm for 3 min. The supernatant was removed, and 75% ethanol was added; then, the sample was centrifuged at 4 °C and 8000 rpm for 3 min. The supernatant was removed, and the RNA pellet was mixed with RNase-free water and transferred to a new Eppendorf tube. The RNA samples were then stored at −80 °C. For the kidney tissue, the samples were ground in liquid nitrogen and homogenized with the TRIzol^®^ reagent. Each liquid sample was mixed with the TRIzol^®^ reagent at room temperature. The RNA extraction process was the same as for the cell line. RNA quantification was performed using a NanoDrop spectrophotometer (Thermo fisher scientific, Waltham, MA, USA).

#### 2.2.4. Protein Extraction

The protein was extracted from the CRFK cells in a 6-well plate. The growth medium was removed, and the cells were washed with PBS. Next, trypsin was applied to detach the cells, and a growth medium was added. The samples were transferred to Eppendorf tubes and centrifuged at 4 °C at 1200 rpm for 10 min. The supernatant was removed, RIPA (lysis buffer) was added, and the samples were left in an ice container. They were then centrifuged at 4 °C at 12,000 rpm for 10 min. The supernatant was collected and stored at −80 °C. Protein quantification was performed using the Bradford assay. The Bradford solution (catalog no. 500-0006) was diluted with PBS. The diluted Bradford solution was then added to the protein samples in 96-well plates. Then, protein lysates were added to each well, and the samples were incubated at room temperature for 10 min. Protein quantification was performed at 540 nm.

### 2.3. Sample Processing

#### 2.3.1. Relative Gene Expression

The total RNA was extracted from the CRFK cells and kidney tissue using the TRIzol^®^ reagent (catalog no. R4533). A real-time polymerase chain reaction was performed using the iQ5 real-time PCR (Bio-Rad, Hercules, CA, USA) instrument with iQ SYBR green supermix (Bio-Rad, Hercules, CA, USA, catalog no. 08-24-00001). The qPCR protocol involved an initial polymerase activation step at 95 °C for 12 min, followed by 40 cycles of denaturation at 95 °C for 15 sec and annealing/extension at 60 °C for 30 s. Data acquisition was performed at the end of the annealing/extension step. All reactions were conducted in triplicate using 96-well reaction plates (2 µL per reaction). In this study, the housekeeping gene was β-actin. The mRNA expression levels of *TGFβ*, *MAPK*, and *Bcl2* were calculated as relative gene expression ratios with β-actin using 2^−∆CT^. The sequences of the primers used in this study are detailed in Table 2.

#### 2.3.2. Western Blot Analysis

Protein samples with a concentration of 30 µL were mixed with 2× Laemmli sample buffer (Bio-Rad, Hercules, CA, USA) containing β-mercaptoethanol and heated at 95 °C. Next, the proteins were separated on 12% SDS-PAGE gels and transferred to a polyvinylidene fluoride (PVDF) membrane with 0.45 µm pores (Bio-Rad), utilizing a Trans-Blot^®^ SD Semi-Dry Transfer Cell (Bio-Rad). Next, each membrane was washed with Tris-buffered saline with 0.05% Tween (TBST) and blocked with BSA at room temperature. The membranes were then incubated with primary antibodies for TGF-β1, MAPK, or β-actin and washed with TBST. Next, they were incubated at room temperature with a secondary antibody labeled as a HRP-conjugated antibody. The primary and secondary antibodies are shown in Table 2. Signal detection was performed using a DAB substrate (Bio Basic, Markham, ON, Canada). Protein quantifying densitometry was performed using Image Studio^TM^ Lite (LI-COR, Lincoln, NB, USA); the protein quantities were then calculated as the ratio of TGF-β1 or MAPK to β-actin as a loading control.

#### 2.3.3. Immunohistochemistry

The kidney tissue of dead cats was collected through necropsy. The tissue was fixed in 10% formalin and embedded in paraffin; it was then sliced into sections with a thickness of 2–7 µm using a rotary microtome. Deparaffinization was conducted using xylene, followed by 100% ethanol and 95% ethanol, before staining. For heat-induced epitope retrieval (HIER), microwave radiation at 800 W was used with a sodium citrate buffer at pH 6. The tissue area was outlined using an immunohistochemistry pen, and 0.3% hydrogen peroxidase (H_2_O_2_) was applied. The slides were washed with PBST. Next, 2.5% BSA in PBS was added to the samples at room temperature, and they were washed with PBST. Immunohistochemical staining was performed by incubating the tissue section with the primary antibodies at 37 °C, followed by washing with PBS. The tissue section was incubated with a dilution of PBS with normal goat serum and washed with PBS. The secondary antibodies were incubated at room temperature in a dark room and washed with PBS. The DAB solution was used to develop the stain at room temperature, followed by washing the slide with tap water and staining with hematoxylin and lithium. The negative control was obtained by substituting the primary antibody with normal mouse serum, following the same protocol. The primary and secondary antibodies are shown in Table 3. The CaseViewer version 2.7.0.191696 program was used to evaluate the level of immunoreactivity.

### 2.4. Statistical Analysis

The data are presented as either the mean ± standard deviation (SD) or median (interquartile; IQR). We assessed their normality using the Shapiro–Wilk test. Then, an unpaired *t*-test was performed to compare the means between the two groups. The Wilcoxon rank sum test was performed on non-parametric data. Each experiment consisted of at least *n* = 4, where *n* was the number of monolayers or cat kidney tissue samples. A *p*-value of less than 0.05 was considered significant.

## 3. Results

### 3.1. Doxorubicin-Induced Cytotoxicity in Feline Kidney Cells

The viability of the CRFK cells was measured after they were treated with DOX at concentrations of 1, 2, 4, and 8 µM for 48, 72, and 96 h. The results revealed that, after 48 h, the cell viability amounted to 83.08%, 76.78%, 64.87%, and 53.83% for the respective DOX concentrations (Figure S1). After 72 h, the cell viability percentages were 111.35%, 97.53%, 107.04%, and 59.37% for the corresponding DOX concentrations (Figure S2). The cell viability after 96 h of treatment was 82.97%, 68.93%, 62.61%, and 61.04% for the respective DOX concentrations (Figure S3). The remainder of the study thus focused on using DOX at a concentration of 8 µM for 48 h.

### 3.2. TGFβ, MAPK, and Bcl2 Relative Gene Expression in Feline Kidney Cells

**Figure 1:**
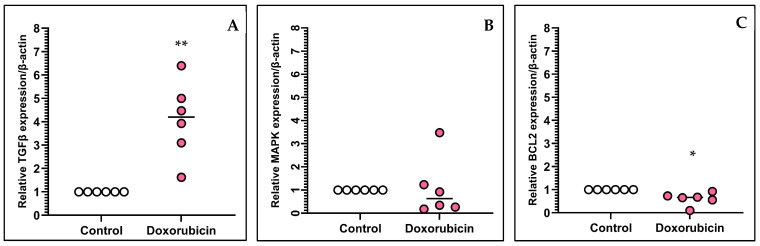
Relative gene expression (fold-change) of *TGFβ* (**A**), *MAPK* (**B**), and *Bcl2* (**C**) in controls and feline kidney cells with DOX-induced toxicity. The horizontal lines indicate the median fold change (gene expression value). * *p* < 0.05, ** *p* < 0.01.

The results revealed that the median fold change in *TGFβ* gene expression in the DOX-treated group was 4.198 (Figure 1A). The *TGFβ* gene expression in the DOX-treated group was significantly higher compared to the control group. The median fold change in *MAPK* gene expression in the DOX-treated group was 0.636 (Figure 1B). The *MAPK* gene expression in the DOX-treated group did not differ from that in the control group. The median fold change in *Bcl2* gene expression in the DOX group was 0.672 (Figure 1C). The *Bcl2* gene expression in the DOX-treated group was significantly lower than that in the control group. The results regarding the *TGFβ*, *MAPK*, and *Bcl2* mRNA expression are presented in Figure 1.

### 3.3. TGFβ, MAPK, and Bcl2 Relative Gene Expression in Kidney Tissues

**Figure 2:**
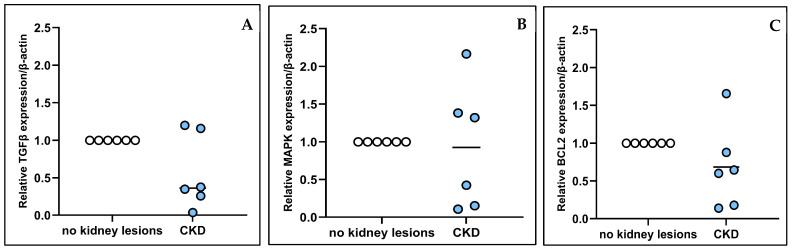
Relative gene expression (fold-change) of *TGFβ* (**A**), *MAPK* (**B**), and *Bcl2* (**C**) in kidney tissue of cats with no kidney lesions and cats with chronic kidney disease (CKD). The horizontal lines indicate the median (**A**) and mean fold changes (**B**,**C**) (gene expression values).

The fold changes in *TGFβ*, *MAPK*, and *Bcl2* gene expression were 0.363, 0.925, and 0.683, respectively (Figure 2A–C). The *TGFβ* and *Bcl2* gene expression in CKD cats was slightly lower than that in cats with no kidney lesions. However, the *TGFβ*, *MAPK*, and *Bcl2* gene expression in the CKD cats was not significantly different from that in the cats with no kidney lesions. The gene expression results for the kidney tissue are shown in Figure 2.

### 3.4. Protein Expression of TGF-β1 and MAPKs in Feline Kidney Cells

The protein expression of TGF-β1 was numerically higher but not statistically different in the doxorubicin-treated group compared to the control group. The protein expression of MAPKs showed a tendency to increase in the doxorubicin-treated group. However, the protein expression of TGF-β1 and MAPKs was not significantly different in the CKD cats compared to the cats with no kidney lesions. The results are shown in Table 4, along with Figures S4 and S5.

### 3.5. Immunohistochemistry of TGF-β1 and MAPKs in Cat Kidney Tissues

**Figure 3:**
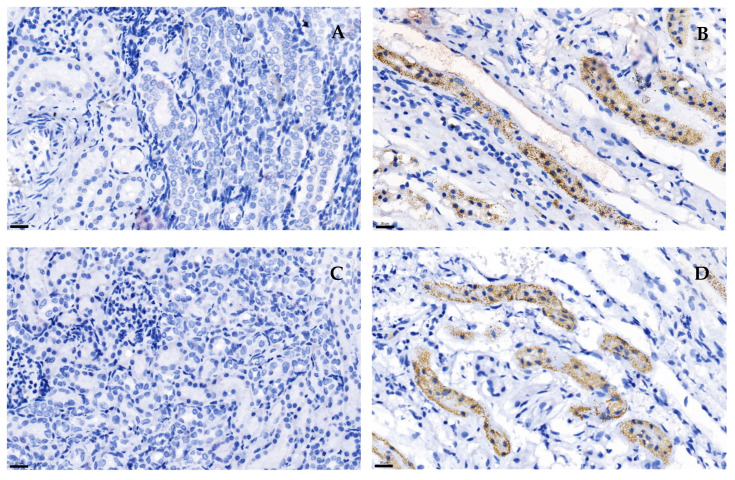
The immunohistochemistry staining of cats’ kidney tissue. TGF-β1 immunohistochemistry staining in a cat with normal kidney tissue (**A**) and a cat with CKD (**B**). MAPK immunohistochemistry staining in a cat with normal kidney tissue (**C**) and a cat with CKD (**D**). In the CKD cat, TGF β1 and MAPK immunostaining were found in the tubulointerstitial area, distal tubules, and collecting ducts (**B**,**D**). Original magnification 400×. Scale bar = 20 µm.

TGF-β1 and MAPK immunostaining was observed in the kidney tissue of CKD cats in the tubulointerstitial area, the intracellular cytoplasmic of distal tubules, and collecting ducts (Figure 3B–D). At the same time, no staining was present in the normal kidney tissue (Figure 3A–C) or the negative control.

## 4. Discussion

The present study investigated the expression of the *TGFβ*, *MAPK*, and *Bcl2* genes in feline kidney cells subjected to doxorubicin-induced cytotoxicity, as well as in the kidney tissue of cats with CKD. The expression of the TGF-β1 and MAPK proteins in doxorubicin-treated feline kidney cells was assessed using Western blot analysis. Additionally, immunohistochemistry was performed to evaluate the staining of the TGF-β1 and MAPK proteins in the kidney tissue of cats with naturally occurring CKD. This study aims to evaluate TGF-β1, MAPK, and Bcl2 gene and protein expression in doxorubicin-induced cytotoxicity in feline kidney cell lines and kidney tissue of cats with CKD. This study hypothesized that doxorubicin-induced cytotoxicity in feline kidney cells and the kidney tissue of cats with CKD would increase levels of TGF-β1 and MAPK while decreasing levels of Bcl-2.

CRFK cells represent an epithelial cell line that was originally derived from the cortex of the feline kidney [22]. CRFK cells have been widely used in studies to assess kidney cell toxicity in cats [23,24,25,26]. However, CRFK cells may not be purely epithelial and could exhibit mesenchymal cells. They have been shown to phenotypically resemble fibroblasts [27]. Primary feline proximal tubular epithelial cells represent a more accurate in vitro model for the investigation of the effects of various insults on the feline renal tubule [28]. However, CRFK cells are the only available cell line derived from feline kidney tissue. Exposure to TGF-β1 has been shown to alter the morphology of CRFK cells, shifting them from a more epithelial phenotype to a more fibroblastic phenotype. This phenotypic change supports the use of CRFK cells as an in vitro model for the study of CKD. Additionally, CRFK cells have been shown to be unsuitable for the direct testing of the effects of angiotensin II (AT-II) on feline renal fibrosis [24], as well as in assessing the impacts of drugs that modulate this pathway, such as DOX [29]. However, the effects of AT-II on renal fibrosis can be evaluated indirectly using CRFK cells, as they exhibit a high level of TGF-β1 [24]. Elevated TGF-β1 expression in CRFK cells has been associated with renal fibrosis, suggesting that these cells can be used to study fibrosis-related mechanisms through the modulation of TGF-β1 [24]. 

Doxorubicin can lead to oxidative stress in glomerular epithelial cells [30] and result in kidney damage [31]. DOX is known to cause chronic nephrotoxicity through an oxidative stress mechanism, which has been suggested to play an important role in DOX nephropathy [32,33]. Similarly, oxidative stress plays a central role in the progression of CKD in cats, with studies showing altered oxidative stress responses in these animals [34]. Oxidative stress is a key factor involved in both DOX-induced nephrotoxicity and CKD in cats. Therefore, DOX is a relevant agent for the induction of cytotoxicity in feline kidney cells, as demonstrated in our in vitro model. An 8 µM dose of DOX, applied for 48 h, was determined to be suitable to induce cytotoxicity. This finding is consistent with a previous study on CRFK cells, which demonstrated a 50% decrease in cell viability when using 8 µM doxorubicin for 48 h [23]. It is also in line with studies on human kidney cells. For example, the cell viability of HK-2 cells decreased significantly when doxorubicin was applied at a concentration of 8 µM for 24 h, dropping to nearly 50% [20,35].

The primary lesion type in CKD cats is tubulointerstitial fibrosis [5]. However, tubular atrophy is also frequently found in the kidneys of CKD cats [12]. Hence, fibrosis and apoptosis are associated with feline CKD development. The main pro-fibrotic mediator associated with renal fibrosis is TGF-β1 [6]. Oxidative stress stimulates TGF-β1 production [36]. TGF-β1 signaling is composed of two pathways: the smad-dependent (canonical) pathway and the smad-independent (non-canonical) pathway [8]. In our study, the expression of the *TGFβ* gene was significantly increased in the DOX-treated group compared to the controls. A previous study on feline kidney cells found that the expression of the genes α-SMA, CTGF, TNC, TSP-1, and COL1 increased following treatment with TGF-β1 [24]. Similarly, in a study on primary feline proximal tubular epithelial cells, it was found that TGF-β1 may be involved in apoptosis and the pathogenesis of renal fibrosis in feline CKD [28]. In feline renal cortical fibroblast cultures, TGF-β1 was implicated in the fibroblast-to-myofibroblast transition, which is a key step in fibrosis [37]. In rat kidney cells, those treated with TGF-β1 showed higher levels of α-SMA, COL1, and COL3 compared to the control [38]. Previous studies on kidney cells have demonstrated the pro-fibrotic mediating effect of TGF-β1 [24,28,37,38]. These findings suggest that TGF-β1 may be involved in the pathogenesis of renal fibrosis in the context of DOX-induced cytotoxicity in feline kidney cells, in line with its involvement in feline CKD.

The present study found that the *TGFβ* gene expression in the kidney tissue of CKD cats was not significantly different from that in cats with normal kidneys. Lourenço et al. (2020) reported significantly higher TGF-β1 transcript levels in the kidneys of CKD cats compared to healthy cats [39]. Similarly, the gene expression of *TGFβ* was higher in the kidney tissue of mice with unilateral ureteral obstruction (UUO) when compared to sham mice [40]. In a study on diabetic nephropathy mice, the *TGFβ* mRNA expression in the kidney tissue of diabetic mice with blood glucose fluctuation (BGF) was the highest compared to diabetic mice without BGF and control mice [15]. These findings suggest that the TGF-β1 gene expression is upregulated in various kidney diseases, including feline CKD. The variability in its expression may be influenced by factors such as the disease stage, sample collection methods, and underlying conditions. In our study, one kidney sample was obtained via nephrectomy, while the others were collected post-mortem. One of the cats with CKD stage III also had polycystic kidney disease (PKD) and severe subcapsular effusion, which may have contributed to the altered TGF-β gene expression in this sample.

In our study, the TGF-β1 protein expression in feline kidney cells tended to increase in the DOX-treated group compared to the control group. The immunohistochemistry analysis of TGF-β1 in the kidney tissue also indicated positive immunostaining in the tubular area in CKD cats. In agreement with a previous study, TGF-β1 immunostaining was predominantly located in the distal tubules and collecting ducts in the kidney tissue of cats with CKD [12]. In contrast, normal feline kidney tissue showed weak immunostaining with TGF-β1 [11]. In another study, kidney tissue from human patients experiencing renal fibrosis showed higher levels of TGF-β and α-SMA immunostaining compared to a negative control [32]. A previous study revealed that the urine TGF-β1-to-creatinine ratio was significantly higher in CKD cats than in healthy ones [10]. Moreover, a moderate correlation was identified between the urine TGF-β1-to-creatinine ratio and interstitial fibrosis [41]. An analysis of the TGF-β1 levels in the blood of cats indicated that the TGF-β1 concentration in those with CKD was significantly lower than that in healthy cats. Furthermore, cats with low circulating TGF-β1 concentrations had shorter survival times [12]. In another previous study, IN-1130 showed potential in reducing renal fibrosis in rats subjected to UUO by inhibiting the TGF-β1 pathway [42]. Therefore, targeting the TGF-β1 pathway is important for the development of therapies aimed at preventing the progression of fibrosis in the kidneys [43].

In our study, we also assessed the *MAPK* gene expression in response to DOX-induced cytotoxicity. The MAPK protein expression in the DOX-treated cells showed a tendency to increase compared to the control cells. MAPKs play a role in the signaling transmission of extracellular stimuli to intracellular responses and apoptosis [16] and oxidative stress [44]. In cardiac cells, phosphorylated p38 MAPK protein expression was significantly increased in DOX-induced H9c2 cardiac cells. However, the total p38 MAPK in DOX-induced cardiac cells did not differ from that in control cells [45]. Moreover, the MAPK signaling pathway can be stimulated by TGF-β through the TAK-1/MKK6/p38MAPK pathway [46], which is categorized as a TGF-β smad-independent pathway [47]. In a previous study, mice subjected to unilateral ischemia–reperfusion injury had higher *TGFβ* and *p38MAPK* mRNA expression than sham mice [48]. Our study also indicated that the MAPK immunoreactivity in the kidney tissue of CKD cats was higher than that in cats with normal kidneys. Several previous studies have revealed a high level of MAPK immunoreactivity in other species with kidney problems [49,50]. In patients with IgA nephropathy, p-p38MAPK immunostaining was predominantly observed in high-grade renal fibrosis [49]. In mouse models, p-p38MAPK immunostaining was prominent in UUO mice [48]. In the context of experimental nephrotic syndrome, p-p38MAPK staining was increased in podocytes, parietal epithelial cells, and glomeruli [50]. Therefore, MAPKs may be associated with feline kidney disease through hypoxia and renal fibrosis, especially the TGF-β1 pathway. In feline PKD, MAPK signaling has been implicated in kidney disease progression [51]. However, our study found no significant differences in the MAPK gene and protein expression between cells with DOX-induced cytotoxicity and control cells or between the CKD and non-CKD groups. Few studies have specifically investigated the MAPK pathway in cats with CKD, so further investigation is warranted. Specifically, the p-p38 MAPK levels should be measured in future studies alongside the total MAPK to better understand its role in feline kidney disease.

In the present study, the *Bcl2* gene expression in the DOX model was significantly lower than that in the controls. However, there was no significant difference in Bcl2 gene expression between the kidney tissue of CKD cats and that of cats with normal kidneys. In a study on experimentally induced apoptosis, the authors observed the downregulation of Bcl-2 and upregulation of p38MAPK and Bax expression [52,53]. In rats with PKD, an increase in Bcl-2 protein expression was observed, while Bcl-XL expression decreased, but Bax protein levels remained unchanged in kidney tissue compared to normal rats [54]. Meanwhile, a study on leukemia cells found that treating them with TGF-β promoted cell death, increased Bax, and decreased Bcl-2 expression [55]. In patients with glomerulonephritis, TGF-β had a negative correlation with Bcl-2 but a positive correlation with Bax [56]. Additionally, the levels of circulating Bcl-2 and TGF-β in cats with CKD were lower than those in healthy cats [12,19]. Bcl-2 was found to be regulated by the TGF-β smad-dependent pathway [47], and it was demonstrated that the downregulation of Bcl-2 may regulate apoptosis in feline CKD [19]. These results suggest that apoptosis is related to kidney cytotoxicity and CKD development in cats. Therefore, further investigation into the Bcl-2 family ratio (including Bcl-2, Bax, and Bcl-XL) should be considered to better understand the regulation of apoptosis in this disease.

Oxidative stress, renal fibrosis, and apoptosis play crucial roles in the progression of feline CKD [6,34,40]. The present study provides information about a TGF-β1 mediator that may be associated with renal fibrosis and apoptosis in feline CKD. This study may lead to further investigations into therapeutic medications that can delay renal fibrosis and apoptosis in CKD and contribute to prolonging the life spans of cats with CKD. However, this study had limitations, including the small number of kidney tissue samples. Increasing the number of tissue samples could provide more valuable information about these mediators.

## 5. Conclusions

The present study found that TGF-β1 was upregulated in feline kidney cells, while Bcl-2 was downregulated. However, there were no significant changes observed in MAPK gene expression in either feline kidney cells or tissue. These findings indicate that TGF-β1 may play key roles in regulating renal fibrosis and the progression of apoptosis in feline kidney cells. Although there have been studies on TGF-β1 inhibitors in various forms of organ fibrosis, research on TGF-β1 inhibitors in renal fibrosis in animals remains limited. Notably, TGF-β1 inhibitors have not been explored in the context of feline CKD. Further research on these mediators could provide critical insights and potentially lead to the development of therapeutic interventions aimed at delaying the progression of CKD in cats.

## Figures and Tables

**Table 1:** The parameters of cats with chronic kidney disease (CKD) and cats with no kidney lesions.

Parameter	Unit	Cats with No Kidney Lesions (*n* = 6)	CKD Cats (*n* = 6)	*p*-Value
Age	years	0.33 ± 0.16	2.54 ± 2.52	0.021 *
Gender				
Male	*n*	2	6	
Female	*n*	4	0	
Breed				
Domestic short hair	*n*	6	6	
BUN	mg/dL	33.45 ± 3.10	28.75 ± 20.90	0.686
Creatinine	mg/dL	0.69 ± 0.30	1.60 ± 1.20	0.027 *
Total protein	g/dL	6.90 ± 3.40	6.35 ± 1.30	0.661
Albumin	g/dL	2.55 ± 0.20	2.72 ± 0.30	0.349
Hematocrit	%	30.00 ± 0.60	28.80 ± 8.10	0.864
WBC	cells/µL	15,870 ± 7000	26,077.50 ± 20,300	0.378

BUN = blood urea nitrogen, WBC = white blood cell, * *p* < 0.05.

**Table 2:** The primer sequences.

Gene Name	Accession	Direction	Sequence	Annealing Temperature (°C)	Size (bp)
*TGFβ*	M38449.1	Forward	CCCTGGACACCAACTATTGC	60	163
		Reverse	TCCAGGCTCCAAATGTAGGG	60	
*MAPK*	XM_003994973.5	Forward	ACTGCTGAGCTAAGACCATGAG	60	119
		Reverse	AAGTCAATGCCACAGTGTGC	60	
*Bcl2*	NM_001009340.1	Forward	CCTATCTGGGCCACAAGTGA	60	123
		Reverse	TAAGAGACCACGGCTTCGTT	60	
β-actin	AB051104.1	Forward	CCATCGAACACGGCATTGT	60	147
		Reverse	TCTTCTCACGGTTGGCCTTG	60	

*Bcl2* = B-cell lymphoma 2, *MAPK* = mitogen-activated protein kinase, *TGFβ* = transforming growth factor-beta.

**Table 3:** Primary and secondary antibodies.

Protein Name	Antibodies	Dilution		Localization
		WB	IHC	IHC
TGF-β1	Primary mouse monoclonal TGF-beta 1 Secondary goat anti-mouse	1:1000 1:5000	1:200	Cytoplasm
MAPK	Primary mouse polyclonal p38 MAPK Secondary goat anti-mouse	1:1000 1:5000	1:200	Cytoplasm
β-actin	Direct-Blot HRP mouse monoclonal anti-β-actin	1:1000		

IHC = immunohistochemistry, MAPK = mitogen-activated protein kinase, TGF-β1 = transforming growth factor-beta1, WB = Western blot.

**Table 4:** TGF-β1 and MAPK protein expression in controls and feline kidney cells with DOX-induced toxicity.

Protein Expression	Control (*n* = 4)	DOX-Treated (*n* = 4)	*p*-Value
TGF-β1	0.02 (0.03)	0.04 (0.06)	0.88
MAPK	0.39 (0.15)	1.45 (3.04)	0.68

DOX = doxorubicin, MAPK = mitogen-activated protein kinase, TGF-β1 = transforming growth factor beta 1.

## Data Availability

Data are contained within the article and Supplementary Materials.

## Supplementary Materials

The following supporting information can be downloaded at: https://www.mdpi.com/article/10.3390/ani15020257/s1.
