# Supplementary material for: Unraveling TGF-β1’s Role in Mediating Fibrosis and Cell Death in Feline Kidney Cells
Source: Animals (Basel). 2025 Jan 17;15(2):257. doi: 10.3390/ani15020257 (PMC11758325; doi:10.3390/ani15020257)
Supplement: Supplementary file 1 [file animals-15-00257-s001.zip › animals-3349229-supplementary.pdf]

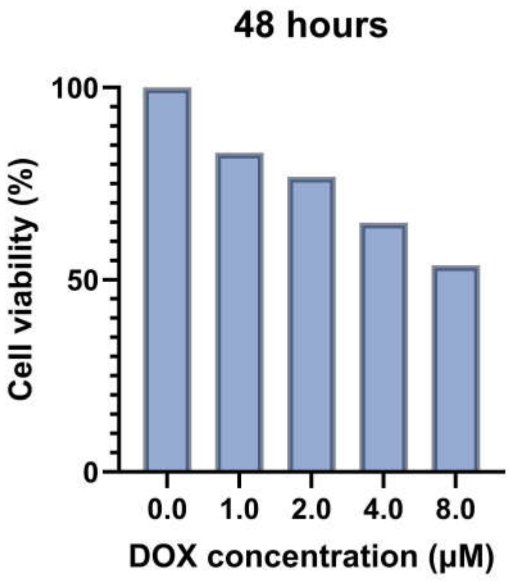

Figure S1: Cell viability of the doxorubicin-treated group in 48 hours

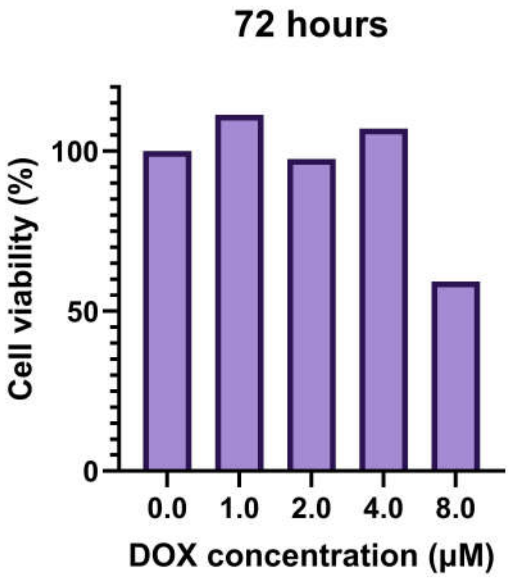

Figure S2: Cell viability of the doxorubicin-treated group in 72 hours

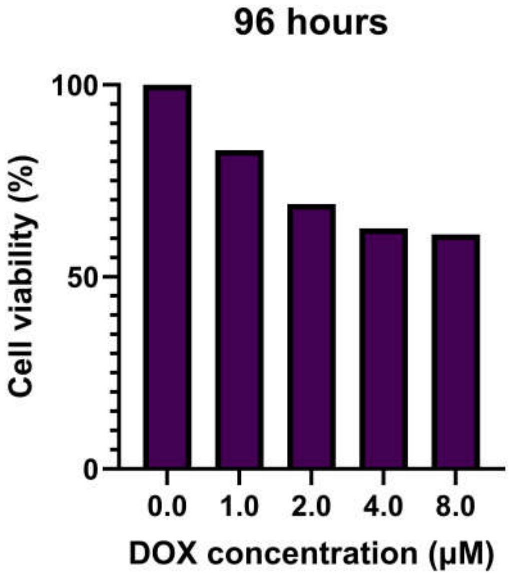

Figure S3: Cell viability of the doxorubicin-treated group in 96 hours

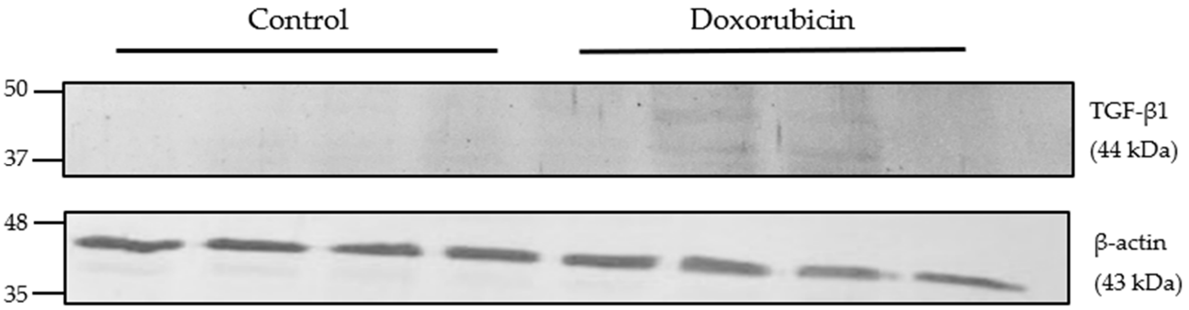

Figure S4: TGF-β and β-actin protein expression via western blot

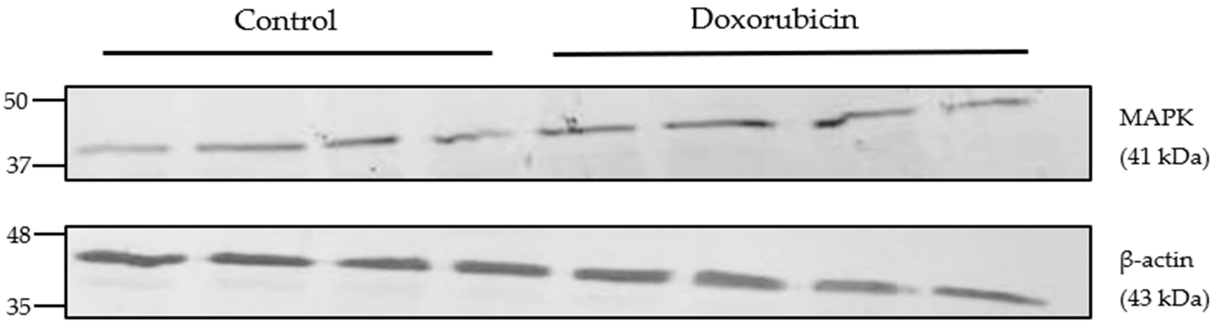

Figure S5: MAPK and β-actin protein expression via western blot
